# A New Surface Decorated Geopolymer Matrix: Insights and Modeling into Comprehensive Sorptive Decolorization Applications

**DOI:** 10.1021/acsomega.6c01379

**Published:** 2026-05-22

**Authors:** Sibel Tunali Akar, Ilknur Kara, Ayse Yilmaz, Tamer Akar

**Affiliations:** a Faculty of Science, Department of Chemistry, 53004 Eskişehir Osmangazi University, 26040 Eskişehir, Türkiye; b Faculty of Education, Department of Elementary Education, 52944 Anadolu University, 26470 Eskişehir, Türkiye; c Graduate School of Natural and Applied Sciences, Department of Chemistry, 53004 Eskişehir Osmangazi University, 26040 Eskişehir, Türkiye

## Abstract

Water contamination caused by synthetic dyes is a severe environmental issue that requires immediate action. Research on treatment and resource conservation technologies is expanding rapidly. One interesting option could be to use environmentally friendly sorbent composites. To efficiently remediate dye-contaminated waters, we manufactured an innovative composite sorbent called MGEOP@MnO_2_ by applying metal oxide particles to the surface of a metakaolin-based geopolymer. Both batch and continuous mode sorption studies verified that MGEOP@MnO_2_ was highly effective at removing the targeted dye. BET, XRD, SEM-EDX, and FTIR techniques were employed to comprehensively evaluate the morphological and structural features of the suggested sorbent, as well as to disclose the possibility of dye interaction. The Box–Behnken design (BBD) and response surface methodology (RSM) were utilized to optimize the process by evaluating the interactive impacts of crucial parameters, including contact time, sorbent amount, initial pH, flow rate, and solution volume. While the D–R, Langmuir, and Freundlich isotherms have significant coefficients of determination and match the experimental data, the pseudo-second-order model dominates the sorption kinetics. After optimizing the settings using the BBD design, the decolorization efficiencies of Bismarck Brown Y (BBY) in batch and continuous systems were 96.71% and 96.14%, respectively. Furthermore, the removal efficiencies of over 90% achieved by MGEOP@MnO_2_ in these systems in the real wastewater application test were remarkable. The results suggest that MGEOP@MnO_2_ could be a viable solution to the water pollution problem caused by dye contamination.

## Graphical abstract

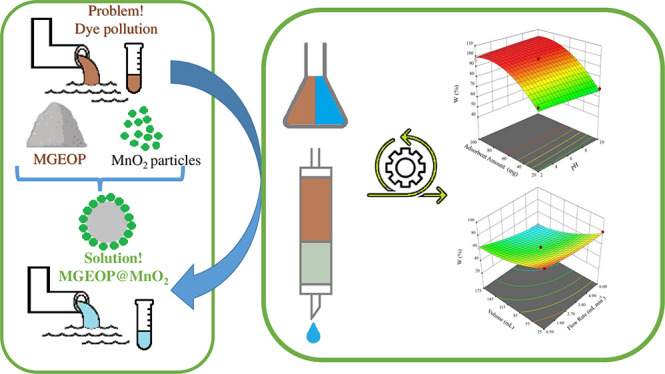

## 1 Introduction

Water resources are being contaminated by a growing number of organic and inorganic contaminants that come from human activity in both industrialized and developing nations. A worldwide issue today is to provide an adequate, safe, and clean water supply in a sustainable manner. In the coming decades, water scarcity might cause everything from war to mass population migration if this is not accomplished. In this context, water from its source to its final point of use must be decontaminated and disinfected using more potent, reliable, and economical operations that do not harm the environment or jeopardize human health throughout the treatment process.^1^

Among various industrial pollutants, synthetic dyes are found to be one of the primary contaminants in wastewater, and their manufacture and implementation are also rather common in today’s world. It is challenging to eliminate these substances from contaminated water due to their light-stable, physiologically inert, and aerobic digestion-resistant structures. They are generated from complicated benzene-based chemicals such diazo and anthraquinone structures. Many of these dyes have been scientifically demonstrated to have high toxicity, carcinogenicity, and mutagenicity.^2–4^ One or more azo bonds (-N = N-) in the structure of azo dyes may be cleaved in metabolic processes, especially by intestinal bacteria and liver enzymes, generating aromatic amines that are frequently linked to hazardous and carcinogenic effects.^5^ Bismarck Brown Y (BBY) is a synthetic azo dye and widely used in industries such as textiles, dyeing, paper, cosmetics, leather, and plastics. Wastewater containing this dye can pose a potential threat to the health of living organisms.^6,7^ Toxicity results from studies using the cationic azo dye BBY on an animal model (*Silurana tropicalis*) show that although this dye is not fatal it causes serious deformities, including a 2-fold increase in p53 and HSP70 mRNA at 1000 mg L^–1^, indicating cellular stress.^8^ One of the main goals of treatment is to remove dyes from the polluted water sources as they have a number of detrimental consequences on aquatic and terrestrial ecosystems, as well as human health.^9^

Typically, contaminated waters are treated using a variety of biological, physical, chemical, and electrical techniques. However, there are many drawbacks to their use, including high operating costs, low selectivity, and operational complexity. Furthermore, removing dye molecules from water using a single treatment procedure is not always feasible. In this regard, utilizing various types of porous adsorbents is an effective option to treatment of contaminated water.^10–14^

In spite of their exceptional performance, the majority of more effective materials (such as metal organic frameworks, activated carbon, etc.) are also costly to utilize and not a practical choice in many regions of the world. As a result, interest has shifted to finding unconventional solid porous materials that have been suggested as inexpensive, effective, environmentally friendly, and outstanding adsorbents for the removal of pollutants.^7^ In this regard, materials that have lately gained attention include geopolymers. These artificial inorganic polymer materials are produced via the reaction of alkaline polysilicate with aluminosilicate oxides to generate Si–O–Al polymeric linkages, usually at temperatures lower than 100 °C.^15^

Geopolymers have various positive properties that make them one of the most popular materials for eliminating contaminants from the aquatic environment. They are also ecologically benign, sustainable, and cost-effective owing to their ease of synthesis. Their porous structures with a large specific surface area allow for the retention of pollutants. The adsorption performance of geopolymers can be improved through various modifications.^16–20^ Many researchers have been working recently to develop superior adsorbents and enhance the adsorption properties of materials. Numerous synthesis techniques have been proposed to address this issue. Decoration of the sorbent surface by various forms of metal oxides was one of the most effective sorbent modification techniques. But, these attempts are still quite limited for the functionalization of geopolymers, which are relatively new types of sorbent materials. Magnetite/geopolymer composite,^21^ magnesium oxide/geopolymer,^22^ TiO_2_/fly ash-based geopolymer,^23^ Fe_3_O_4_/clay-based geopolymer^24^ are some of the notable recent dye remover agents reported in this context.

Among metal oxides, manganese dioxide (MnO_2_) is the most favorable due to its low cost, high stability and surface area, structure flexibility, environmental compatibility, and toxic pollutant affinity aspects. It has a variety of crystallographic planes, layers, and tunnels that provide ample space to harmful contaminants. As such, MnO_2_ is gradually becoming popular as an adsorbent and an efficient sorbent modifying agent.^25–27^ Nevertheless, some drawbacks to using MnOx directly as an adsorbent include the particles’ tendency to self-aggregate, the difficulty of solid–liquid separation, and the leaching of nanoparticles with treated effluent. One practical solution to get around these restrictions is to anchor particles into supporting materials.^28^

This study suggests a new and efficient hybrid sorbent based on coating the geopolymer matrix surface with MnO_2_, taking into account the properties of both metal oxides and geopolymers. In addition to successfully functionalizing the geopolymer structure, the collaborative effect of MnO_2_ and a metakaolin-based geopolymer also eliminates the negative aspects of metal oxides. The novelty of this study not only encompasses the design of a new modified geosorbent for dye removal, but also the generation of useful data for developing a water treatment model. For this purpose, the simultaneous effects of critical sorption parameters were investigated for BBY adsorption using the proposed sorbent material, both in batch and column systems, using a Box-Behnken quadratic design based on the Response Surface Methodology (RSM).^29^ The industrial sector benefits greatly from this design^30^ since it enables quick prediction and adjustment to the constantly shifting effluent conditions.

In conclusion, this study is a concise investigation that offers information on the synthesis of MnO_2_-decorated geopolymer material (MGEOP@MnO_2_), its potential for dye removal in both batch and continuous systems. The simultaneous effects of critical sorption parameters, the clarification of the process-related interactions using various instrumental analyses including BET, XRD, SEM-EDX, and FTIR and the practical application of the suggested material were examined. We hope that this unified strategy will be useful promote environmental cleanup initiatives.

## 2 Material and Methods

### 2.1 MGEOP@MnO_2_ Preparation and Dye Solution

The geopolymer based on metakaolin was prepared by combining it with an alkaline activator, as we previously reported.^31^ Briefly, a mechanical stirrer was used to dissolve NaOH (99%) in a sodium silicate solution with a SiO_2_/Na_2_O molar ratio of 2:1. Metakaolin (Mefisto L05) (52.9% SiO_2_, 41.9% Al_2_O_3_, 1.8% TiO_2_, 1.1% Fe_2_O_3,_ and 0.2% MgO) was added to this solution by stirring at 1000 rpm for 15 min. Following synthesis, the geopolymer slurry was put into HDPE containers and left to cure at 80 °C for 48 h. After being cured, the materials were ground using an IKA A11 laboratory mill and sieved through a 212-μm sieve. Distilled water was used to wash away the geopolymer substance. Excess alkali was eliminated from the geopolymer by thoroughly washing it with distilled water. It was then dried for 3 h at 60 °C in an MMM Medcenter Ecocell model oven. After combining 25 g of geopolymer with a 500 mL of 0.07 M MnCl_2_.2H_2_O aqueous solution, the MnO_2_-decorated geopolymer (MGEOP@MnO_2_) was prepared by adding 250 mL of 0.1 M KMnO_4_ solution dropwise under continuous stirring until the precipitation was completed according to the following equation.

$$3{\mathrm{Mn}}^{2+}+2{{\mathrm{MnO}}_{4}}^{-}+2H_{2}O\to 5{\mathrm{MnO}}_{2}↓+4H^{+}$$ 1

The MGEOP@MnO_2_ was dried once more at 80 °C for 2 days following being separated from the reaction medium using filter paper and washed with distilled water. It was stored in closed glass bottles.

The model pollutant BBY dye (C_18_H_18_N_8_.2HCl, C.I. 21000) was acquired from Sigma-Aldrich. One gram of BBY was dissolved in one liter of distilled water to prepare the dye stock solution. By diluting the stock sample, the experimental solutions were prepared. Analytical-grade reagents and deionized water were used to prepare all of the solutions utilized in this work. 0.1 M NaOH and/or 0.1 M HCl solutions were used to adjust the pH of the aqueous solutions.

### 2.2 Characterization Studies and Apparatus

Instrumental analysis is a useful tool for figuring out which characteristics-structural, morphological, optical, and/or physicochemical-control the adsorption capacity and for clarifying the responsible mechanisms when the dyes interact with the adsorbents.^32^

The BET-specific surface areas of the geopolymer, MGEOP@MnO_2_, and dye-loaded MGEOP@MnO_2_ were assessed with a TouchWin surface area analyzer from Quantachrome Instruments. pH values were recorded using a pH meter (WTW-Inolab 720). A scanning electron microscope (SEM - Zeiss SUPRA 50 VP) and FESEM, Hitachi Regulus, equipped with an energy-dispersive X-ray detector was used to analyze the surface morphology and elemental composition of the adsorbent, respectively. Functional groups on the surface of the adsorbent were determined through IR spectral analysis (Bruker Tensor 27 FTIR spectrophotometer) at the wavenumber range of 4000–400 cm^–1^. Zeta potential measurements were performed using a Zetasizer Nano ZS (Malvern Instruments) to assess the surface charge characteristics of MGEOP@MnO_2_. Additionally, the Malvern Mastersizer 2000 laser diffraction particle size analyzer was used to evaluate the particle size distribution of the sorbent material.

### 2.3 Experimental Sorption Design

Experimental design is a statistical method for enhancing the planning and running of experiments. By using this method, researchers can find the variables that affect trial outcomes by methodically organizing and controlling them. As a result, it requires fewer experiments, minimizes errors, and offers accurate, thorough information. Recent research has shown that this approach is frequently and successfully used in both industrial and scientific research.^33,34^

Both the variables that will be studied and the limit values of the predefined variables are taken into consideration by the relevant program during the experimental design process. The equation that follows is used to determine the total number of experiments (*N*):

$$N=2k^{*}\left(k-1\right)+c_{p}$$ 2

The factor number is represented by *k*, while the number of replicates at the center point is denoted by *c*_p_. Design-Expert software (version 13.0) was used to develop and randomize the experimental design. A total of 17 experimental runs, with three repetitions at the center point, were conducted for both batch and continuous modes. The regression model’s relevance was evaluated using model fit tests and analysis of variance (ANOVA). In order to determine the factors impacting the dye removal effectiveness of MGEOP@MnO_2_, three parameters were investigated in both batch and continuous systems. Tables 1 and 2 contain comprehensive details on the experiments and related responses.

**Table 1:** Box–Behnken Experimental Design for BBY Adsorption onto MGEOP@MnO_2_ in Batch System

			**variables limits**			
**variables**	**symbol**		low (−1)	center (0)	high (+1)	**ΔX**_**i**_
pH	A		2	6	10	4
*m* (mg)	B		20	60	100	40
*t* (min)	C		5	40	75	35
Experimental and predicted values for BBY removal process in batch system						
**run order**	**level of variables**			**BBY removal (%W)**		
	A	B	C	observed	predicted	residual
	pH	m (mg)	t (min)			
1	2	60	5	85.84	83.72	2.12
2	2	20	40	71.12	68.57	2.55
3	2	100	40	96.70	99.31	–2.62
4	2	60	75	93.92	95.96	–2.04
5	6	20	5	42.24	46.90	–4.66
6	6	100	5	95.59	95.09	0.50
7	6	60	40	97.42	97.37	0.05
8	6	60	40	97.46	97.37	0.09
9	6	60	40	97.41	97.37	0.04
10	6	60	40	96.54	97.37	–0.83
11	6	60	40	98.03	97.37	0.66
12	6	20	75	75.20	75.70	–0.50
13	6	100	75	98.99	94.33	4.66
14	10	60	5	84.14	82.09	2.04
15	10	20	40	68.68	66.06	2.62
16	10	100	40	99.59	102.13	–2.55
17	10	60	75	95.77	97.89	–2.12

**Table 2:** Box–Behnken Experimental Design for BBY Adsorption onto MGEOP@MnO_2_ in Continuous System

			**variables limits**			**ΔX**_**i**_
**variables**		**symbol**	low (−1)	center (0)	high (+1)	
flow rate (mL min^–1^)		A	0.5	3.25	6	2.75
m (mg)		B	20	60	100	40
V (mL)		C	25	100	175	75
Experimental and predicted values for BBY removal process in continuous system						
**run order**	**level of variables**			**BBY removal (%W)**		
	A	B	C	observed	predicted	residual
	(mL min^–1^)	m (mg)	V (mL)			
1	3.25	60	100	59.23	57.50	1.73
2	3.25	60	100	59.30	57.50	1.80
3	3.25	60	100	53.04	57.50	–4.46
4	3.25	60	100	55.73	57.50	–1.77
5	3.25	60	100	60.19	57.50	2.69
6	3.25	20	25	54.61	55.00	–0.39
7	3.25	100	25	87.37	90.04	–2.67
8	3.25	20	175	26.25	23.58	2.67
9	3.25	100	175	56.90	56.51	0.39
10	6	20	100	27.75	29.47	–1.72
11	6	100	100	58.67	58.10	0.57
12	6	60	25	80.25	78.15	2.10
13	6	60	175	38.52	39.47	–0.95
14	0.5	60	25	90.76	89.80	0.96
15	0.5	60	175	61.43	63.53	–2.10
16	0.5	100	100	83.03	81.31	1.72
17	0.5	20	100	41.41	41.97	–0.56

### 2.4 Batch and Continuous Mode Adsorption Procedure

BBY adsorption experiments were conducted in a batch system using 25 mL of BBY solution at a concentration of 100 mg L^–1^. Different amounts of MGEOP@MnO_2,_ ranging from 20 to 100 mg, were mixed with the dye solution using a Variomag Poly 15 magnetic stirrer at 260 rpm. Following the adsorption process, the solid and liquid phases were successfully separated by centrifugation for three minutes at 4000 rpm.

Adsorption studies in a continuous system were performed using glass columns packed with synthesized sorbent material between glass wool layers, and experimental details are included in Table 2. A peristaltic pump (Ismatec Ecoline) was used to pass the dye solution through the column.

After all adsorption experiments, a Shimadzu UV-2550 spectrophotometer was utilized to measure the concentrations of BBY in the aqueous phase at a maximum wavelength of 457 nm.

Each adsorption experiment was conducted three times, and the means are used to calculate the adsorption capacity (*q*_e_) and removal efficiency (%) of the adsorbent by eqs 3 and 4;

$$q_{e}=\frac{V\left(C_{i}-C_{e}\right)}{m}$$ 3

$$\mathrm{Removal}\;\mathrm{efficiency}\;\left(\%\right)=\frac{\left(C_{i}-C_{e}\right)}{C_{i}}\times 100$$ 4

Where *C*_i_ and *C*_e_ are the initial and equilibrium BBY concentrations (mg L^–1^), respectively. *V* is the BBY solution volume (L), and *m* is the MGEOP@MnO_2_ mass (g).

The SPSS 17.0 program was used to perform the statistical analysis. The standard errors are shown by the error bars in the figures.

### 2.5 BBY Sorption Kinetics and Isotherm Modeling

The kinetics study provides an in-depth knowledge of the adsorbent-pollutant interaction depending on experimental conditions. The Lagergren pseudo-first-order model (eq 5),^35^ Ho and McKay pseudo-second order model (eq 6),^36^ and Weber-Morris (eq 7)^37^ intraparticle diffusion model were employed to describe the kinetics of BBY sorption onto MGEOP@MnO_2_.

$$\ln\left(q_{e}-q_{t}\right)=\ln q_{e}-k_{1}t$$ 5

$$\frac{t}{q_{t}}=\frac{1}{k_{2}q_{e}^{2}}+\frac{1}{q_{e}}t$$ 6

$$q_{t}=k_{p}t^{1/2}+C$$ 7

In these equations, *q*_e_ represents the adsorption capacity at equilibrium (mg g^–1^), *q*_t_ indicates the amount of BBY adsorbed on MGEOP@MnO_2_ at time *t* (mg g^–1^), *k*_1_ represents the pseudo-first-order reaction rate constant (min^–1^), the pseudo-second-order reaction rate constant is denoted by *k*_2_ (min^–1^), *k*_p_ is intraparticle diffusion rate constant (mg g^–1^ min^–1/2^) and *C* is the intercept, obtained by extrapolation of the linear portion of plot.

Adsorption isotherms are mathematical models that describe how a substance’s amount adsorbed onto an adsorbent surface varies with its equilibrium concentration. The adsorption of BBY dye onto the MGEOP@MnO_2_ was evaluated using Langmuir,^38^ Freundlich,^39^ and Dubinin–Radushkevich (D-R)^40^ isotherm models. The equations of these isotherms are presented in eqs 8 to 10:

$$\mathrm{Langmuir};q_{e}=\frac{q_{\max}K_{L}C_{e}}{1+K_{L}C_{e}}$$ 8

$$\mathrm{Freundlich};q_{e}=K_{F}C_{e}^{1/n}$$ 9

$$D-R;\ln q_{e}=\ln q_{m}-\beta \varepsilon^{2}$$ 10

where *q*_e_ (mol g^–1^) refers to the amount of BBY adsorbed at equilibrium, *q*_max_ (mol g^–1^) is the maximum monolayer adsorption capacity of MGEOP@MnO_2_. *C*_e_ (mol L^–1^) indicates the BBY concentration at equilibrium. The constant *K*_L_ (L mol^–1^) represents the Langmuir adsorption constant. *K*_F_ ((mol g^–1^) (mol L^–1^)^−1/n^) denotes the Freundlich adsorption constant, and *n* (dimensionless) is a constant related to the adsorption intensity. β (mol^2^ J^–2^) is related to the average free energy of adsorption per mole of adsorbate, *q*_m_ (mol g^–1^) denotes the theoretical saturation capacity, and ε (mol kJ^–1^) represents the Polanyi potential.

The equation below can be used to calculate the average free energy of adsorption (*E*, kJ mol^–1^).

$$E=\frac{1}{{\left(2\beta \right)}^{1/2}}$$ 11

*E* value indicates the type of adsorption occurring: if *E* is less than 8 kJ mol^–1^, it signifies physical adsorption; if 8<*E* < 16 kJ mol^–1^, it indicates chemical ion exchange; and if *E* is greater than 16 kJ mol^–1^, it reflects chemical adsorption. Higher *E* values indicate stronger interactions between the adsorbate and adsorbent. Additionally, the D-R isotherm provides insight into the distribution of adsorption energies and the porosity of the adsorbent material.

### 2.6 Application Test in Real Wastewater

A real wastewater sample was provided from a textile factory (Bursa, Turkey) to test the adsorbent’s practical applicability. The wastewater was spiked with a known quantity of BBY dye to achieve a final concentration of 100 mg L^–1^ as the actual wastewater did not contain BBY. Using real wastewater, the dye removal capabilities of MGEOP@MnO_2_ were examined in both batch and continuous systems.

## 3 Results and Discussion

### 3.1 Modeling of the BBY Adsorption onto MGEOP@MnO_2_

A three-factor Box-Behnken experimental design^29^ was employed to optimize the process parameters and examine the impact of important factors on both the batch and column adsorption processes. The observed and predicted values of the BBY adsorption efficiency, considering both real and coded factor variables, are presented in Tables 1 and 2. These data were also utilized for process optimization. ANOVA results for BBY adsorption using MGEOP@MnO_2_ are shown in Tables S1 and S2. Equations 12 and 13 display the regression equations derived from the data fitting and regression analysis.

$$W_{\mathrm{batch}}=97.37+0.08A+16.70B+7.01C+1.33\mathrm{AB}+0.89\mathrm{AC}-7.39\mathrm{BC}-0.72A^{2}-12.63B^{2}-6.74C^{2}$$ 12

$$W_{\mathrm{column}}=57.50-8.93A+16.99B-16.24C-2.68\mathrm{AB}-3.10\mathrm{AC}-0.53\mathrm{BC}+3.34A^{2}-8.12B^{2}+6.91C^{2}$$ 13

Both the adsorbent amount and contact time significantly affected the adsorption of BBY by MGEOP@MnO_2_ in the batch system (*p* < 0.05). In contrast, the BBY removal was not significantly influenced by the solution’s initial pH (*p* > 0.05). Furthermore, the significance of the suggested statistical model is indicated by the model values being <0.0001. The data in Table S2 clearly showed that the BBY adsorption of MGEOP@MnO_2_ in the continuous system is significantly influenced by the flow rate, adsorbent amount, and solution volume (*p* < 0.05).

The evaluation of the model was based on the predicted adsorption yield values of BBY presented in Tables 1 and 2. BBY adsorption onto MGEOP@MnO_2_ in batch and column systems was effectively described by the quadratic regression model, as evidenced by the findings, which showed an appropriate agreement between the observed and predicted values. In this study, the *R* ^2^ values of 0.9769 for the batch system and 0.9880 for the column system affirm that the regression model provides accurate estimates of BBY adsorption efficiency. The model’s reliability was further supported by the fact that *F* values for both systems were significant (*p* < 0.0001).

The relationship between the experimental and predicted results for the batch and column systems, respectively, is clearly seen in Figure 1a,b. With *R* ^2^ values of 0.9769 and 0.9880 for the batch and column systems, respectively, these figures show a good correlation, highlighting the ability of the suggested model to precisely approximate the actual values. Notably, the higher *R* ^2^ value for the continuous system suggests an enhanced predictive capability for BBY removal on MGEOP@MnO_2_, making it a promising approach for further investigation and application.

**Figure 1:**
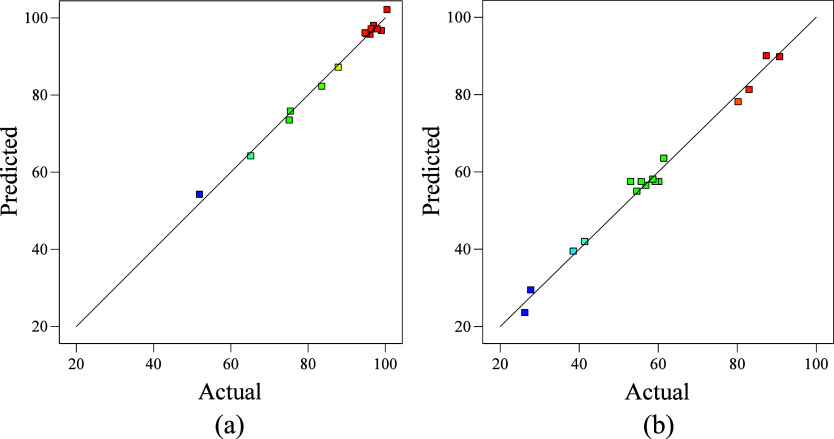
Plots of actual vs predicted BBY removal yield (%) of MGEOP@MnO_2_ in batch (a) and continuous (b) systems.

### 3.2 BBY Adsorption Performance of MGEOP@MnO_2_ as Function of Model Parameters

#### 3.2.1 Effects of Batch System Interactive Variables on BBY Removal

The impacts of batch processing variables (pH, adsorbent amount, and contact time) on the BBY adsorption efficiency of MGEOP@MnO_2_ were examined using 3D response surface plots, and the plots were presented (Figure 2a–c). Figure 2a depicts the interactive influence of the MGEOP@MnO_2_ dosage and initial pH on the sorption process. This figure revealed that initial pH values between 2.0 and 10.0 had no impact on the removal of BBY and that MGEOP@MnO_2_’s dye adsorption capability remained nearly constant. A sorption effectiveness that is stable throughout this pH range is linked to the negative surface charge of geopolymer-based sorbent materials, which can be stable over a broad pH range. The study on the removal of Basic Blue 7 from aqueous media using a metakaolin-based geopolymer showed a similar sorption trend.^41^ The good removal performance throughout a broad pH range is a significant advantage for the environmental and economic aspects of MGEOP@MnO_2_’s practical applications, as it does not necessitate the addition of acid or base to modify the ambient pH. Increasing the amount of MGEOP@MnO_2_, on the other hand, resulted in a better adsorption efficiency. The amount of adsorbent enhances adsorption because of the larger surface area and the availability of more adsorption sites at higher concentrations.^42^ This finding also concurs with those documented in the literature.^43^ On the other hand, Figure 2b demonstrates the impact of varying initial pH values and contact time on the sorption yield of the MGEOP@MnO_2_ for BBY molecules. The figure reveals that increasing contact time has a favorable impact on sorption efficiency rather than a synergistic effect. This observation can be explained by the fact that extended interaction periods allow dye molecules to engage more effectively with the active sites on MGEOP@MnO_2_, as expected.

**Figure 2:**
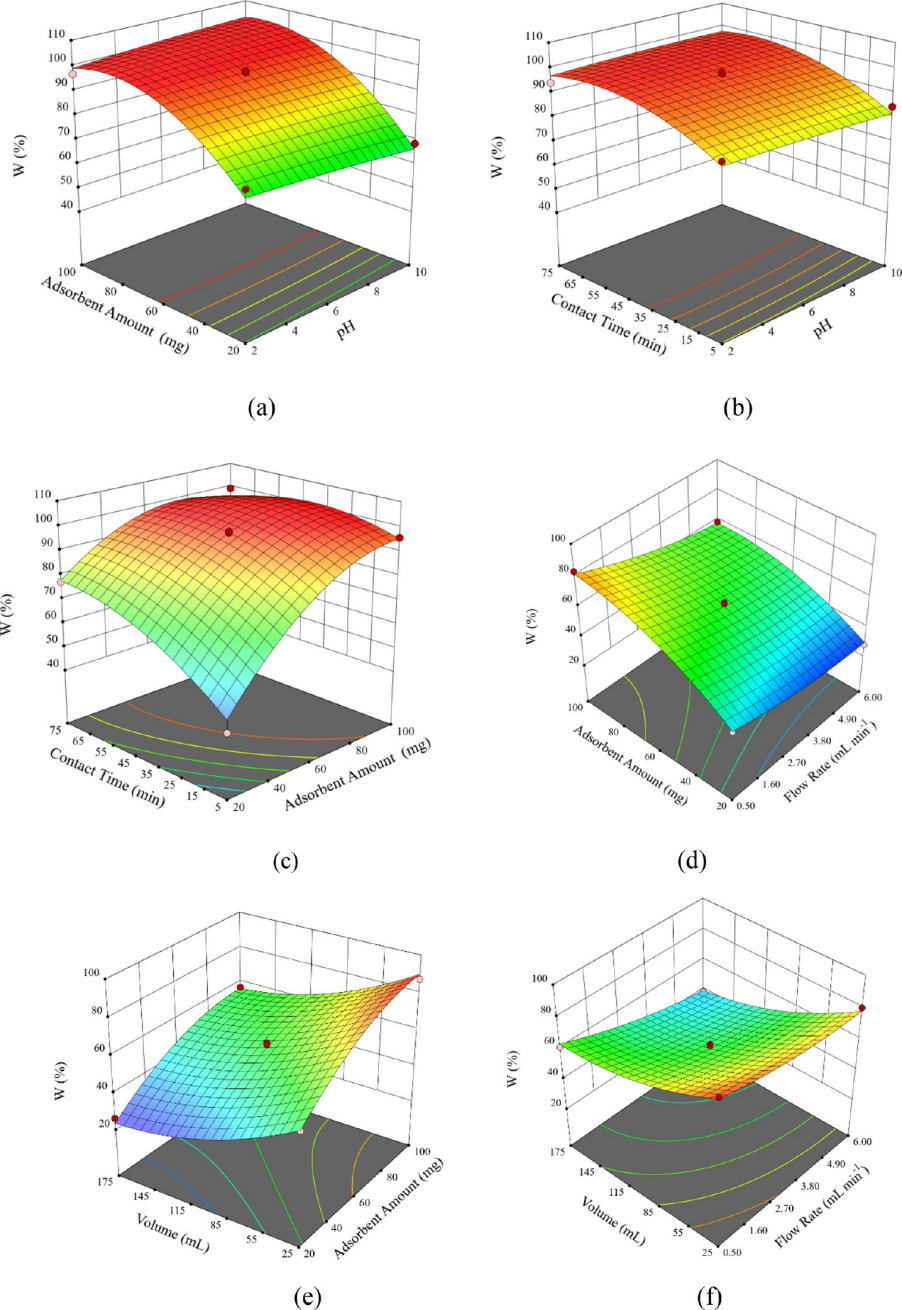
3D response surface plots for the interactive effects of pH and adsorbent amount (a) pH and contact time (b), adsorbent amount and contact time (c) on BBY removal yield (%) of MGEOP@MnO_2_ in batch system; and 3D response surface plots for the interactive effects of flow rate and adsorbent amount (d), adsorbent amount and solution volume (e), and flow rate and solution volume (f) on BBY removal yield (%) of MGEOP@MnO_2_ in continuous system.

Figure 2c shows how MGEOP@MnO_2_ dosage and contact time affect the sorption process of BBY dye. The 3D surface plot shows a convex curvature, indicating that the sorption yield increases significantly with increasing MGEOP@MnO_2_ dosage and contact time, implying a synergistic interaction. In other words, the highest sorption efficiency was obtained with the maximum sorbent amount and the longest contact time. A higher removal efficiency results from an increase in MGEOP@MnO_2_ dose because more active binding sites and surface area are available. In like manner, as contact time increases, the interaction between dye molecules and MGEOP@MnO_2_ increases, resulting in improved removal efficiency.

#### 3.2.2 Effects of Column System Interactive Variables on BBY Removal

The effects of continuous mode sorption processing variables (flow rate, sorbent amount, and solution volume) on the BBY sorption efficiency of MGEOP@MnO_2_ were also analyzed using 3D response surface plots. The combined effects of the column system’s factor variables are depicted in Figure 2d,e. Figure 2d shows the 3D surface plot of the interactive effect of flow rate and sorbent dosage on the BBY removal yield of MGEOP@MnO_2_. As shown in this figure, increasing the MGEOP@MnO_2_ dosage resulted in a higher sorption yield, peaking at a 0.5 mL min^–1^ flow rate. Figure 2e shows the relationship between MGEOP@MnO_2_ dosage and sorbate volume. The 3D surface plot reveals that the maximum decolorization yield was observed at lower sorbate volume and when a higher amount of adsorbent was used. The combined impact of flow rate and solution volume on the removal of BBY in the continuous system was depicted in Figure 2f, where the lowest flow rate and the lowest solution volume generated the maximum dye removal yield. The contact time between MGEOP@MnO_2_ and the dye molecules increases at low flow rates because the dye solution takes longer to move through the column. As film diffusion and intrapore diffusion resistances drop, dye molecules are better able to access active binding sites. The small amount of solution improved the sorption performance more significantly. The MnO_2_-coated geopolymer’s adsorption ability can handle the limited total dye load entering the column at low solution volumes, which causes the adsorbent surface to saturate later. As a result, the % elimination values increase. However, the adsorbent binding sites rapidly become saturated as a result of the increased total dye amount at higher volumes, which causes an early rise in dye concentration at the column outlet. The literature also included reports of similar findings.^44,45^

### 3.3 Optimization

A desirability bar chart summarizing the ideal solution set for the BBY dye adsorption using MGEOP@MnO_2_ is shown in Figure 3. The desirability values, ranging from 0 to 1.000, display the overall suitability of the studied experimental parameter combinations for maximizing the target responses. The efficiency of variables such as pH, adsorbent amount, contact time, flow rate, and adsorbate volume in reaching the best adsorption outcomes is shown graphically in this chart. Values nearer 1 indicate the best conditions in both systems. These ratings show that the chosen optimization settings match the goals of optimizing BBY sorption. The efficiency of RSM optimization process is highlighted in this visualization, which effectively balanced operational parameters and adsorption performance to improve dye removal efficiency while using less material and operating for shorter periods of time.^46^ In summary, the optimum batch adsorption conditions for BBY removal by MGEOP@MnO_2_ were determined as pH = 6.0, amount of adsorbent = 60 mg, and contact time = 40 min (Figure 3a). Column system parameters were optimized as flow rate of 2.05 mL min^–1^, dye solution volume of 29.78 mL, and adsorbent mass of 92.43 mg (Figure 3b). The responses for maximum BBY adsorption onto MGEOP@MnO_2_ were estimated to be 97.37% and 93.87% for batch and column systems, respectively, at the determined optimum adsorption conditions. These findings closely correspond to the experimental results, which reported 96.71% and 96.14% for the batch and column systems, respectively. Overall, the optimal conditions for maximum BBY adsorption onto MGEOP@MnO_2_ in both batch and column systems were accurately predicted using the Box-Behnken experimental design, demonstrating good agreement with the experimental findings.

**Figure 3:**
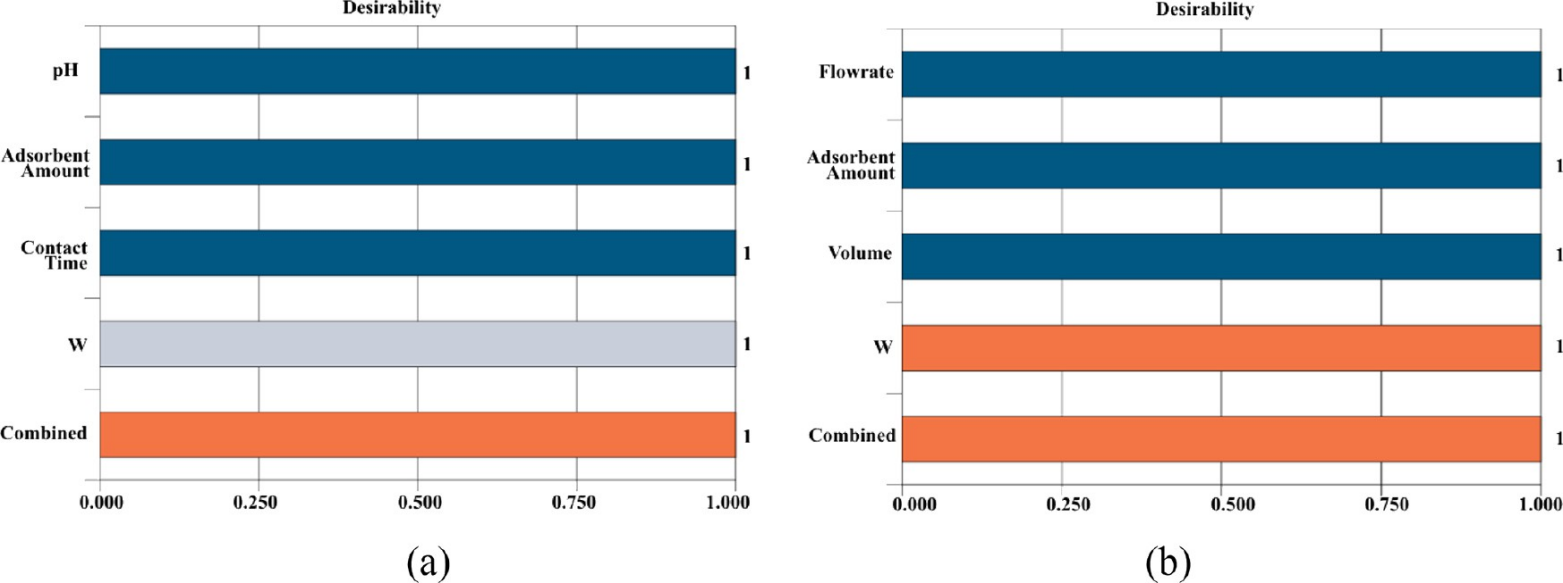
Desirability graphs for the optimization of selected parameters in batch (a) and continuous (b) systems.

### 3.4 BBY Sorption Kinetics of MGEOP@MnO_2_

An experimental investigation using batch adsorption was also conducted to investigate the mechanism of the BBY adsorption process, incorporating changes in contact duration (5–75 min) while keeping a constant initial dye concentration of 100 mg L^–1^, at optimal MGEOP@MnO_2_ dose and pH. Experimental data regarding dye sorption kinetics were analyzed using pseudo-first-order and pseudo-second-order models. The kinetic plots and the related kinetic parameters are presented in Figure 4 and Table 3. The coefficient of determination (*R* ^2^) value for the pseudo-first-order kinetic model was determined to be extremely poor, and the calculated adsorption capacity (*q*_e_) value did not align with the experimental results. This finding indicates that the pseudo-first-order model is not suitable for explaining the BBY adsorption process onto MGEOP@MnO_2_. On the other hand, the pseudo-second-order kinetic model yielded a high *R* ^2^ value of 0.9997, and the calculated *q*_e_ value of 41.36 mg g^–1^ was quite similar to the experimental value of 40.44 mg g^–1^. This good match indicates that chemical interactions are the main factor controlling the adsorption process.^47^ Recently, an identical kinetic mechanism for color removal utilizing a sorption technique has been documented for a variety of composite sorbents.^48–51^

**Figure 4:**
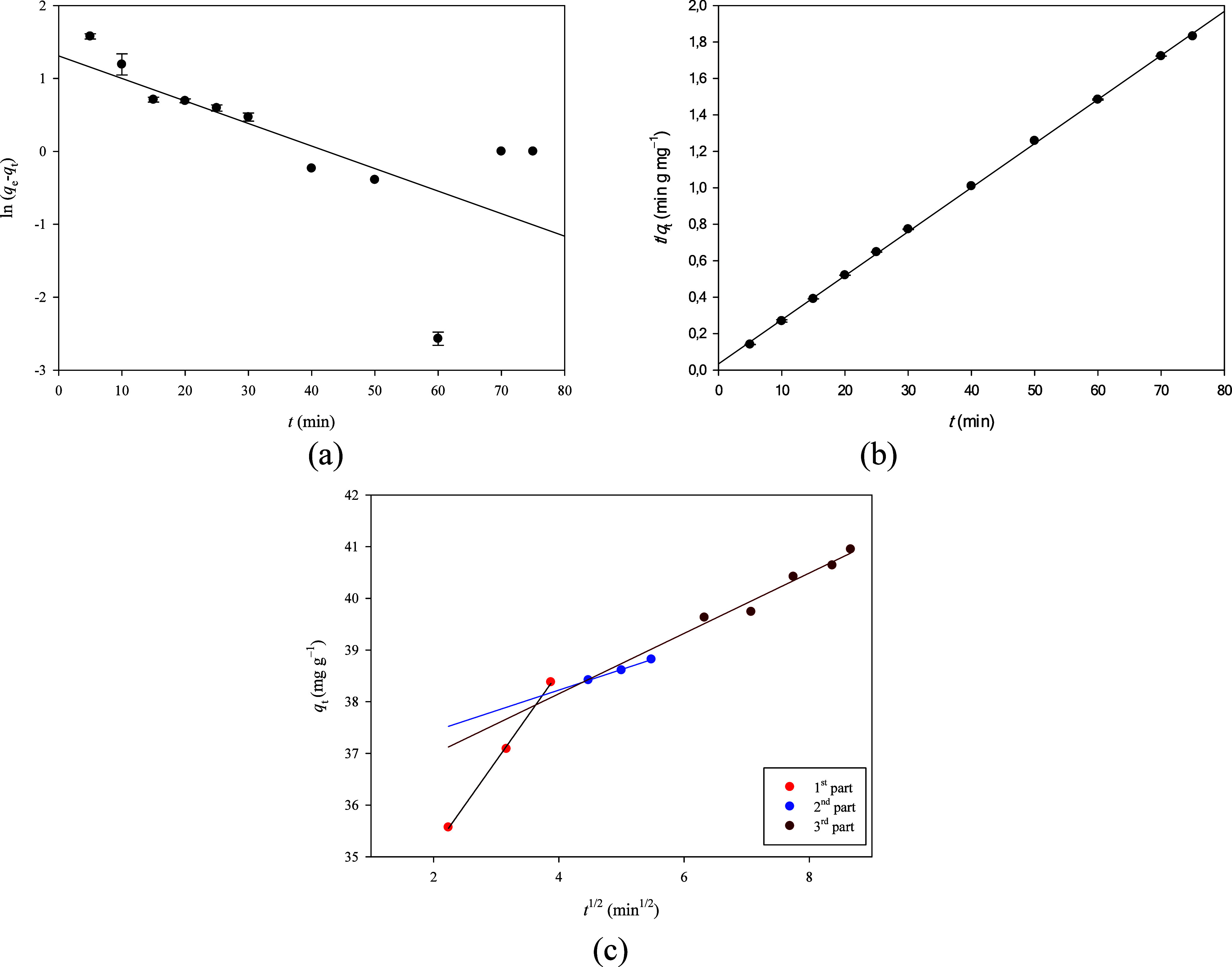
Pseudo-first-order (a) and pseudo-second-order (b) kinetic plots and intraparticle diffusion plot (c) for BBY adsorption onto MGEOP@MnO_2_.

**Table 3:** Kinetic Parameters for the Adsorption of BBY onto MGEOP@MnO_2_

pseudo-first-order kinetic model		
*k*_1_ (1 min^–1^)	*q*_*e*_ (mg g^–1^)	*R* ^2^
3.34 × 10^–2^	0.37	0.8490

pseudo-second-order kinetic model		
*k*_2_ (g mg^–1^ min^–1^)	*q*_*e*_ (mg g^–1^)	*R* ^2^
1.70 × 10^–2^	41.36	0.9997

intraparticle diffusion^a^		
*k*_p_ (mg g^–1^min^–1/2^)	*C* (mg g^–1^)	*R* ^2^
3.98 × 10^–1^	36.63	0.9968

a 2^nd^ part results.

The sorption process was also evaluated by the intraparticle diffusion model. The findings show that boundary layer diffusion, intraparticle diffusion, and equilibrium were the three different stages of the adsorption process (Figure 4c). If the plot of *q*_t_ versus *t* ^1/2^ shows a straight line passing through the origin, then intraparticle diffusion is the single component governing the adsorption process. However, if the findings display multilinear graphs, the adsorption is influenced by two or more processes. In Figure 4c, the second part of plot (*R* ^2^ = 0.9968) represents micropore diffusion but intraparticle diffusion cannot completely regulate the sorption process since none of the lines cross zero.

### 3.5 Adsorption Isotherms for BBY Removal Process

In order to comprehend the interaction between BBY molecules and MGEOP@MnO_2_, as well as dye uptake capability, the experimental equilibrium data were analyzed using Langmuir, Freundlich, and Dubinin–Radushkevich (D-R). Isotherm model plots are presented in Figure 5 while the parameters for these models are included in Table 4. The (D-R) isotherm shows the best match for the adsorption process; however, all isotherm models have *R* ^2^ values over 0.96. The Langmuir model also well describes the adsorption process, having a *R* ^2^ value of 0.9725. Therefore, dye molecules bind to the adsorbent surface in a monolayer at uniform energy levels.^52,53^ The calculated monolayer adsorption capacity of the developed adsorbent is 6.51 × 10^–4^ mol g^–1^ (272.97 mg g^–1^). The energy value (*E*) of 14.69 kJ mol^–1^, calculated from the D-R isotherm, suggests that chemical ion exchange could play an essential role in the adsorption mechanism. The *n* value of the Freundlich isotherm, which is higher than 1, adsorption process is considered to occur spontaneously, and the adsorbent – adsorbate interactions are strong and effective.^54,55^

**Figure 5:**
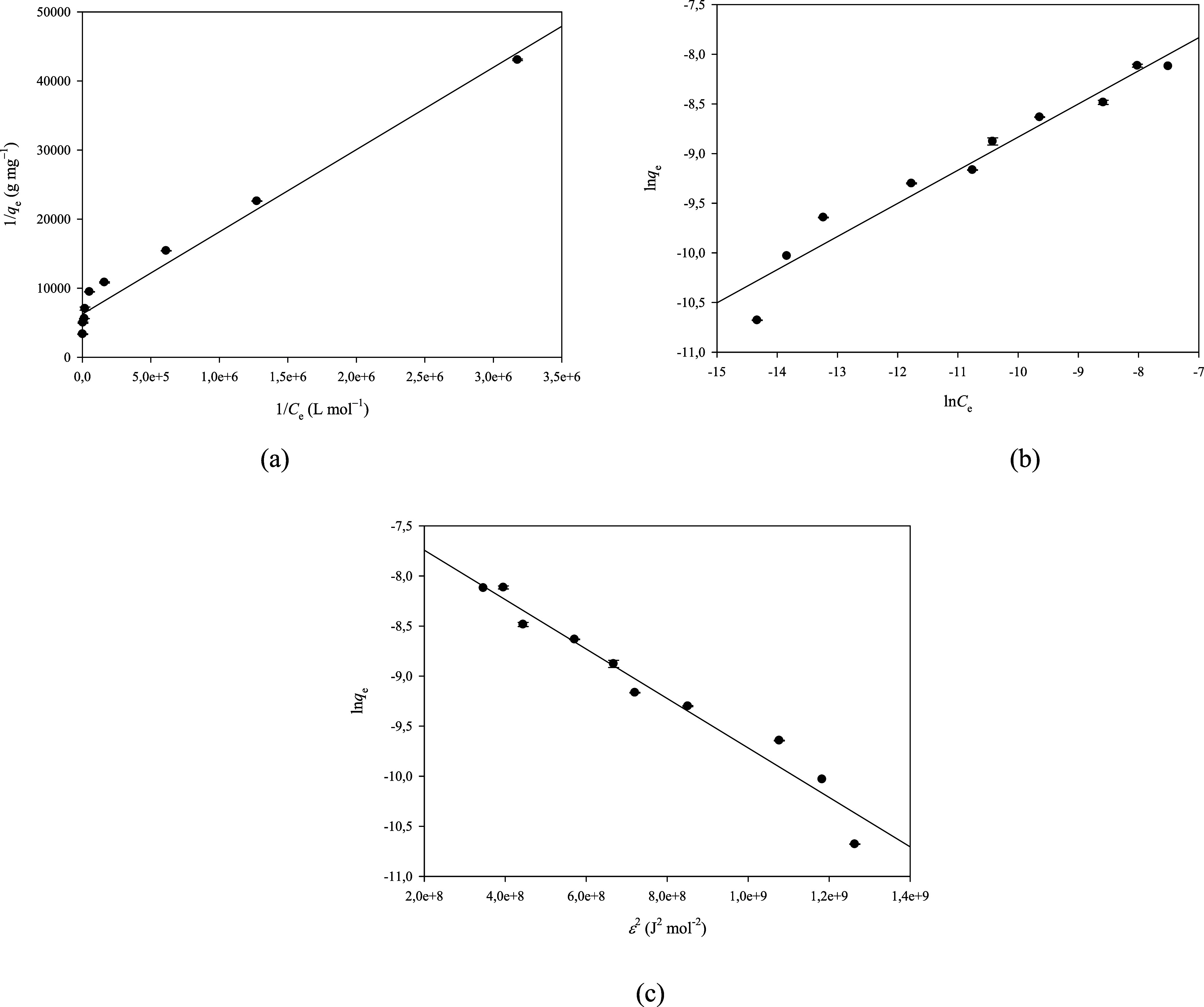
Langmuir (a), Freundlich (b), and D-R (c) isotherm plots for the adsorption of BBY onto MGEOP@MnO_2_.

**Table 4:** Isotherm Parameters for the Adsorption of BBY onto MGEOP@MnO_2_

Langmuir			Freundlich		
*q*_max_ (mol g^–1^)	*K*_L_ (L mol^–1^)	*R*_L_^2^	*n*	*K*_F_ ((mol/g) (mol/L) ^–1/n^)	*R*_F_^2^
1.62 × 10^–4^	5.29 × 10^5^	0.9725	3.23	3.34 × 10^–3^	0.9607

Dubinin–Radushkevich (D–R)					
*q*_max_ (mol g^–1^)	β (mol^2^kJ^–2^)		R_*D*–*R*_^2^	*E* kJ mol^–1^	
6.51 × 10^–4^	2.32 × 10^–9^		0.9774	14.69	

### 3.6 Real Wastewater

The use of the proposed composite sorbent, MGEOP@MnO_2_, to remove BBY dye from real samples is an important aspect of assessing the method’s practical applicability. MGEOP@MnO_2_’s decolorization performance in optimized batch and column systems was tested using an actual wastewater sample from a textile plant that was spiked with 100 mg L^–1^ BBY. Absorption spectra in Figure 6, showed that the intensity of the peak at 457 nm significantly decreased in the spectrum of wastewater after BBY adsorption process. In addition, BBY removal yields of the MGEOP@MnO_2_ in real wastewater were 90.79% and 92.22% in batch and continuous systems, respectively. A slight reduction in BBY removal efficiency under real wastewater conditions could be due to the complex composition of textile industry wastewater. The MGEOP@MnO_2_ synthesized in this study, however, has the important advantage of demonstrating highly effective decolorization performance even in real wastewater conditions.

**Figure 6:**
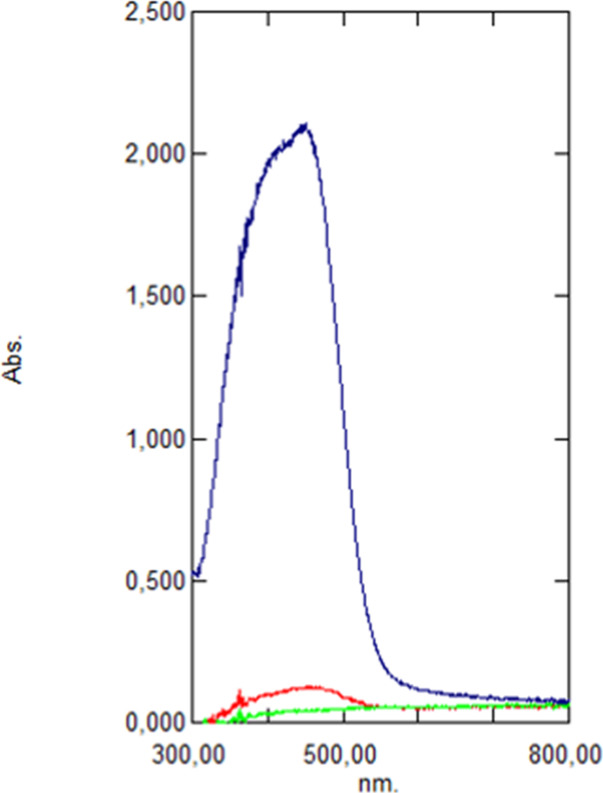
UV/vis spectra of real wastewater before and after BBY adsorption onto MGEOP@MnO_2_.

### 3.7 Characterization

The XRD findings displayed that metakaolin-containing geopolymers exhibited two types of diffraction patterns, which matched to crystalline materials found in metakaolin as impurities (quartz, anatase, muscovite, etc.) and aluminosilicate amorphous materials.^56^ Figure 7 shows the XRD spectra of the MGEOP@MnO_2_ proposed in the study before and after dye loading, as well as the XRD spectrum of the metakaolin-based geopolymer employed as the starting material. These spectra in Figure 7 appear to be consistent with the specified XRD pattern. Furthermore, analysis of the spectra revealed that the MnO_2_ coating and subsequent adsorption process did not result in any structural alterations. The MGEOP@MnO_2_ exhibits an amorphous structure with a small amount of quartz, an impurity resulting from the metakaolin used in the geopolymer synthesis.^57^

**Figure 7:**
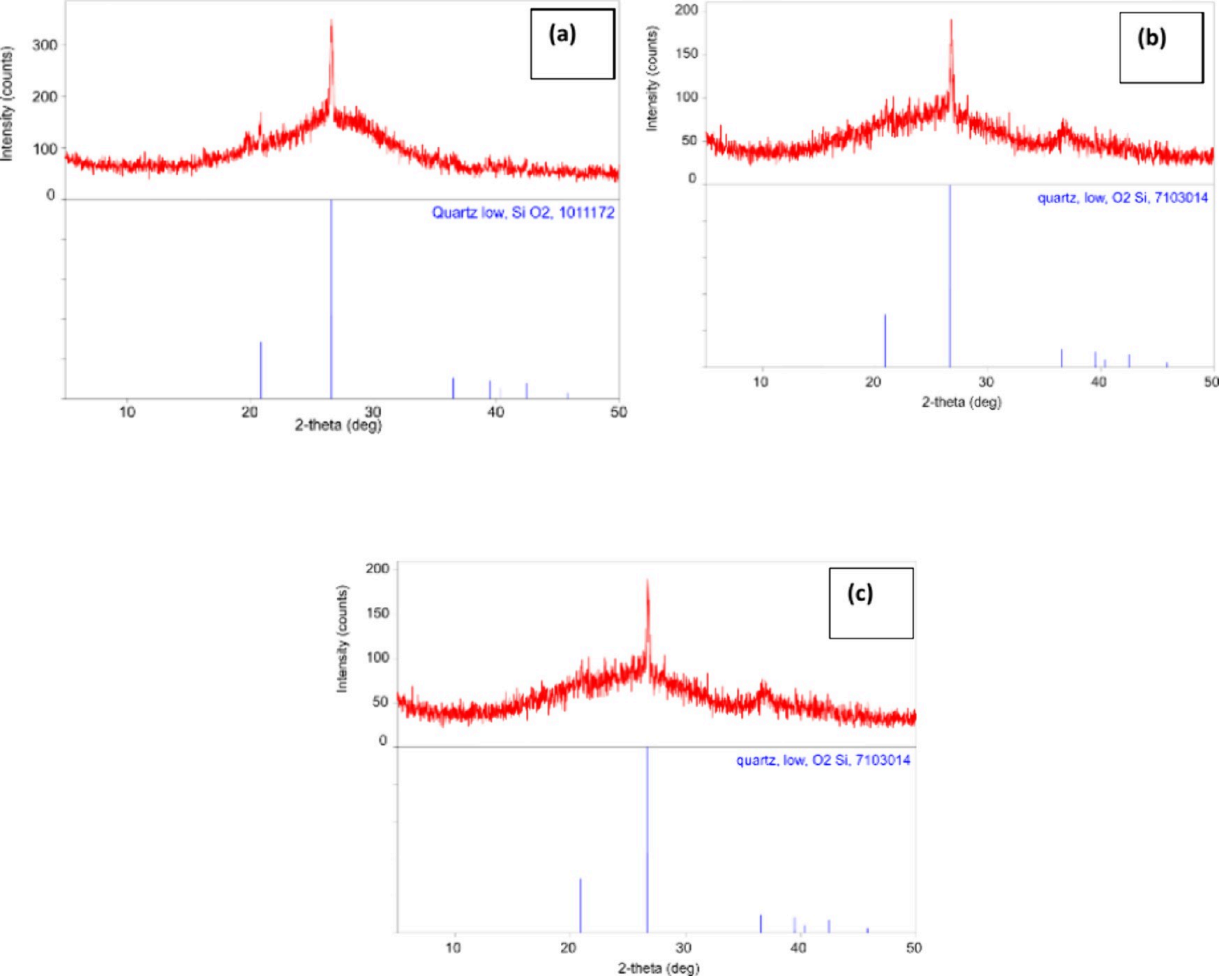
XRD graphs of geopolymer (a), MGEOP@MnO_2_ (b), BBY-loaded MGEOP@MnO_2_ (c).

The zeta potential-pH curve of MGEOP@MnO_2_ is shown in Figure 8a. At low pHs, the surface charge of MGEOP@MnO_2_ is positive. An increase in the pH of the solution causes a negative surface charge, and at strong basic conditions, the adsorbent’s surface becomes more negatively charged. ZPC of MGEOP@MnO_2_ adsorbent was determined as pH 3.30.

**Figure 8:**
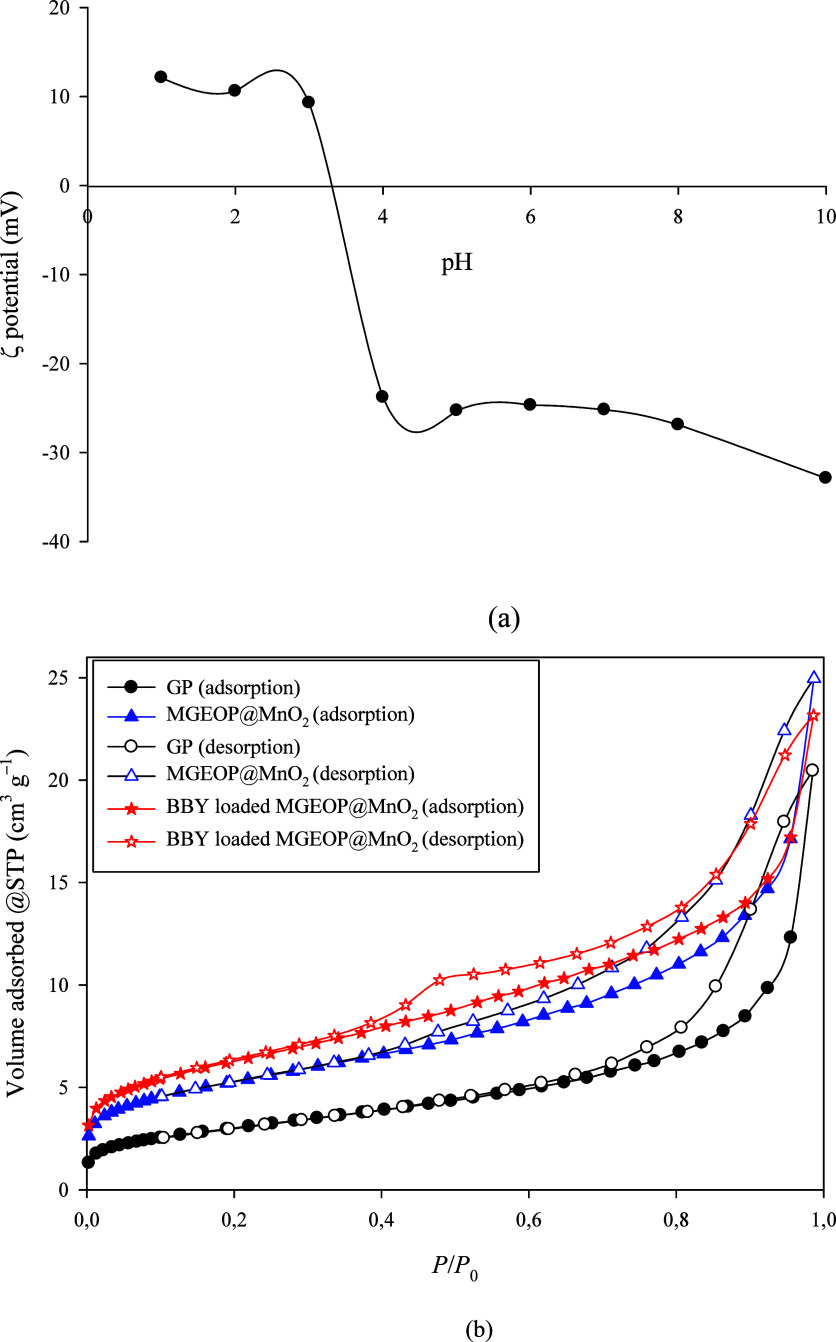
Zeta potentials as a function of the pH of MGEOP@MnO_2_ (a), Multipoint BET N_2_ adsorption–desorption isotherms of GP, MGEOP@MnO_2_, and BBY loaded MGEOP@MnO_2_(b).

The specific surface area and pore characteristics of precursor geopolymer and MGEOP@MnO_2_ were measured using BET analysis. The BET results in Table 5 revealed significant differences in the surface and pore properties of geopolymer and MGEOP@MnO_2_. Both the surface area and pore area of the MGEOP@MnO_2_ increased significantly from 57.59 to 103.95 m^2^ g^–1^ and from 2.658 to 103.95 m^2^ g^–1^, respectively. The pore radius of the geopolymer also increased from 1.58 to 2.09 nm after being coated with MnO_2_. This increase observed after modification in the values of the surface area and pore characteristics of the precursor material has been reported similarly in the literature after the synthesis of biochar and MnO_2_ composite material.^58^ Figure 8b shows the N_2_ adsorption–desorption isotherms for GP, MGEOP@MnO_2_ and BBY-loaded MGEOP@MnO_2_. N_2_ adsorption isotherms of MGEOP@MnO_2_ and BBY-loaded MGEOP@MnO_2_ may be categorized as Type III, which corresponds to macroporous materials, according to the IUPAC classification.

**Table 5:** BET Analysis Results of Geopolymer and MGEOP@MnO_2_

	surface area	pore area	pore radius	pore volume	total pore volume
material	(m^2^ g^–1^)	(m^2^ g^–1^)	(nm)	(cm^3^ g^–1^)	(cm^3^ g^–1^)
geopolymer	57.59	2.658	1.58	6.60 × 10^–4^	0.153
MGEOP@MnO_2_	103.95	17.353	2.09	6.93 × 10^–3^	0.183

Scanning electron microscopy (SEM) images in Figure 9a,b illustrate that the geopolymer powder is composed of irregularly broken grains ranging in size from 10 to 100 μm, with an average grain size of 27 μm (Figure 10). At higher magnifications (Figure 9b), geopolymer precipitates of approximately 30 nm in size are visible.

**Figure 9:**
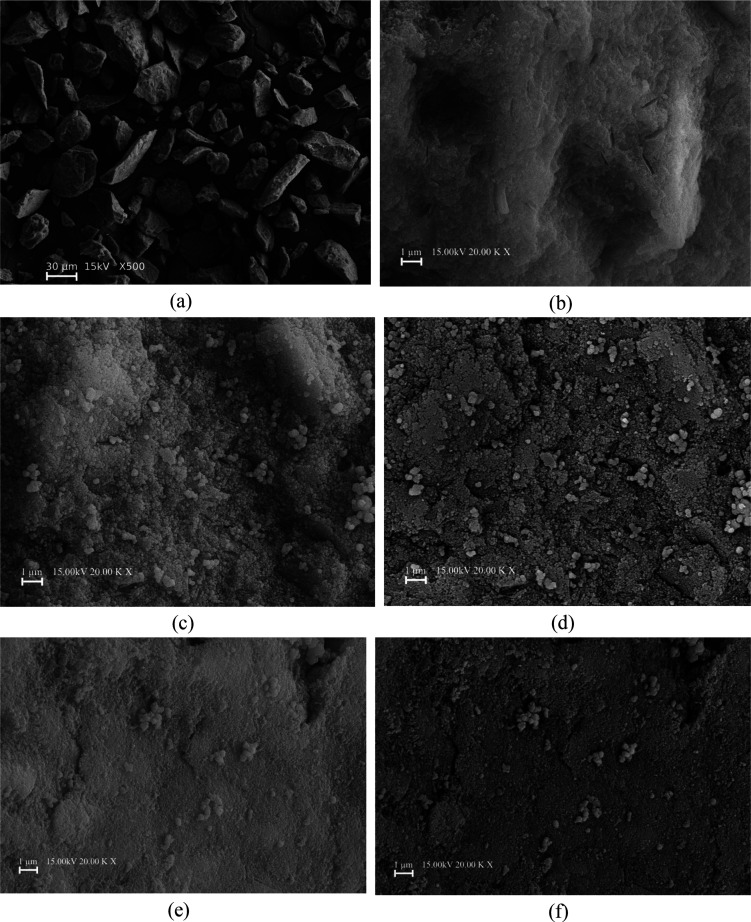
SEM (a) and enlarged SEM images of geopolymer (b); electron (c) and backscattered electron (d) images of MGEOP@MnO_2_; secondary electron (e) and backscattered electron (f) images of the MGEOP@MnO_2_ particle surface after the BBY adsorption.

**Figure 10:**
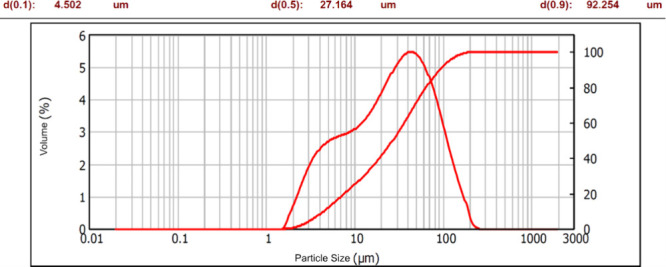
Particle size distribution of geopolymer.

The secondary electron and backscattered electron images of the MGEOP@MnO_2_ are presented in Figure 9c,d). As shown in both photographs, the nanogeopolymer precipitates have become more distinct. A small number of additional precipitated grains, approximately 0.2 to 0.3 μm, are visible on the surface. The energy-dispersive X-ray (EDX) analysis indicates that these grains are manganese-rich, confirming that they are MnO_2_ precipitates formed during the process. Furthermore, most of the manganese has penetrated the geopolymer structure. After the dye adsorption process, the images of the particle surface, including secondary electrons and back-reflected electrons, are shown in Figures 9e,f, respectively. No significant change was observed in the surface morphology following the adsorption process, and the geopolymer precipitates resembled those of the MGEOP@MnO_2_.

Si, Al, and Na are the major elements of the geopolymer structure (Figure 11a). The EDX analysis of MGEOP@MnO_2_ (Figure 11b) showed a manganese peak in addition to these major elements. Figure 9d also shows that the absence of MnO_2_ precipitates in the geopolymer grain indicates that manganese is homogeneously distributed in the geopolymer structure in ionic form. EDX spectrum of dye-loaded adsorbent in (Figure 11c) indicated that the quantity of manganese decreased after the dye was introduced into the sorbent structure. Additionally, the EDX spectra of MGEOP@MnO_2_ showed no N and Cl signal before BBY adsorption, but these peaks were clearly visible at about 0.5 and 2.6 keV following the adsorption process. This observation provides another evidence for the adsorption of BBY azo dye onto MGEOP@MnO_2_.

**Figure 11:**
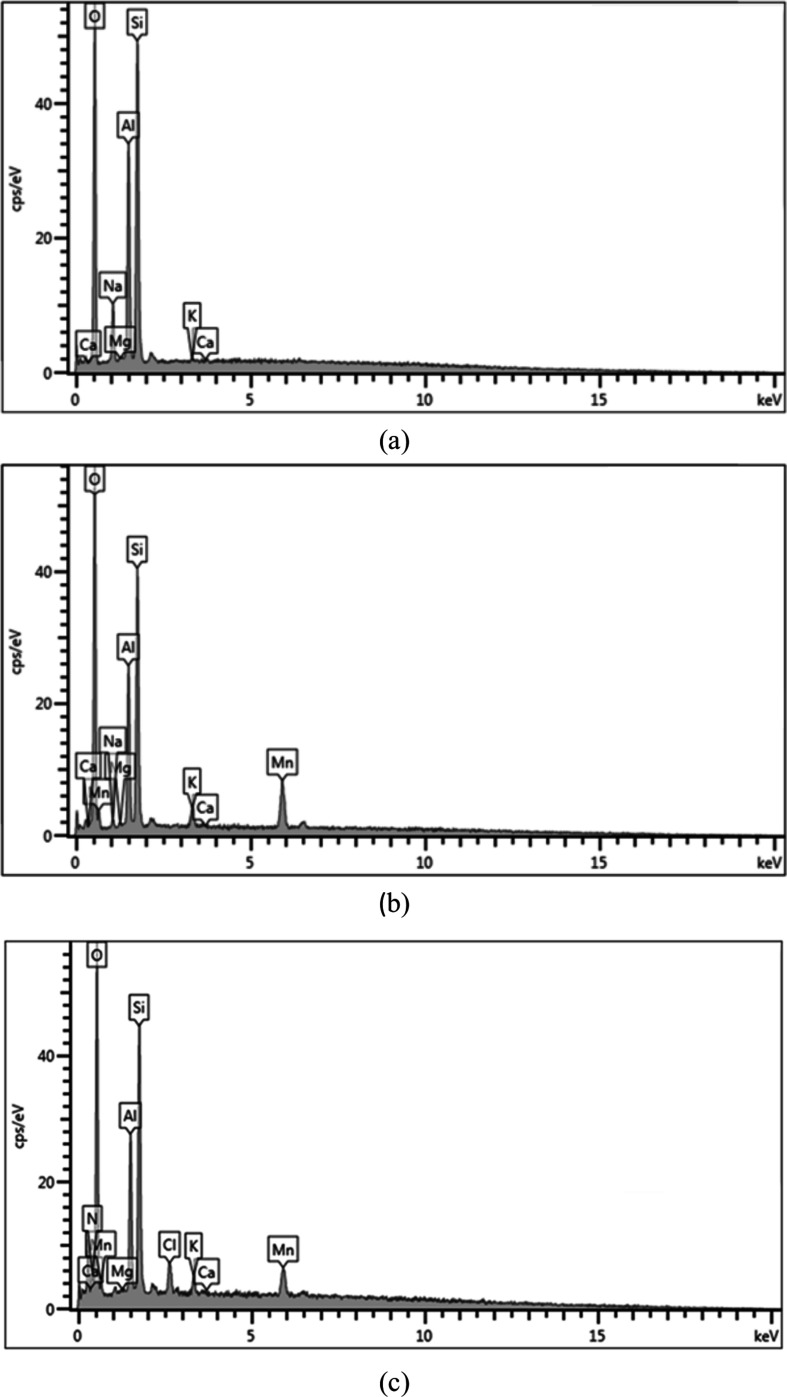
EDX spectra of the geopolymer (a), MGEOP@MnO_2_ (b), and BBY-loaded MGEOP@Mn*O*_2_(*c*).

Figure 12 illustrates the FTIR spectra of the geopolymer, MGEOP@MnO_2_, and BBY-loaded MGEOP@MnO_2_. The broad bands in the FTIR spectrum of geopolymer in the range of 3200–3600 cm^–1^, indicating the presence of hydroxyl (−OH) groups and adsorbed water. The peaks around 1650 cm^–1^ indicate H–O–H bending vibrations, whereas those between 1000 and 1100 cm^–1^ imply Si–O–Si stretching vibrations. The geopolymer’s spectrum shows peaks in the range of 500–720 cm^–1^ due to Al–O–Si bending vibrations whereas the peak at 435 cm^–1^ is caused by Si–O and Al–O bending vibrations.^16,59^ Coating the surface with MnO_2_ caused noticeable changes in the geopolymer’s spectrum. The peak around 3440 cm^–1^ shifted to approximately 3410 cm^–1^, whereas the peak around 1020 cm^–1^ moved to 1035 cm^–1^. Additionally, a dominant peak related to Mn–O stretching vibrations appears at roughly 450–530 cm^–1^. These results indicate that the manganese dioxide coating process alters the surface of the sorbent material. Similar changes caused by MnO_2_ coating on the sorbent material surface have also been reported in the literature.^31,60,61^ After the decolorization process, some changes were also observed in the spectrum of the MnO_2_ coating material. The peaks around 3410 cm^–1^ shifted to 3400 cm^–1^, but the intensity of the Mn–O peak slightly decreased. These findings further confirm the interaction between the MGEOP@MnO_2_ and BBY dye.

**Figure 12:**
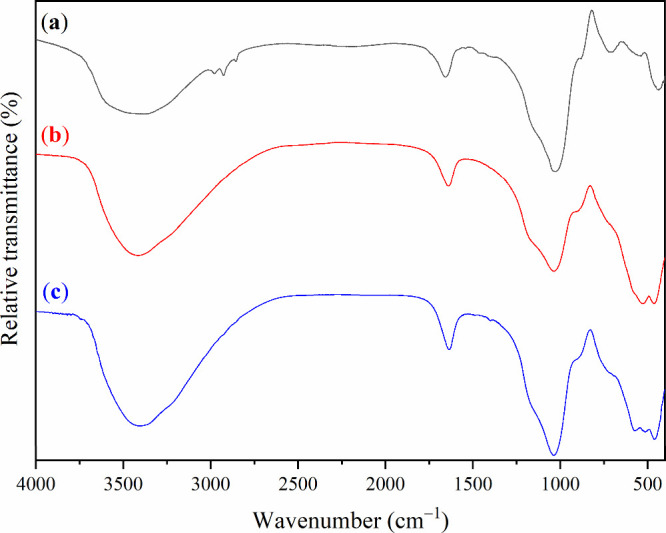
FTIR spectra of geopolymer (a), MGEOP@MnO_2_ (b), and BBY-loaded MGEOP@MnO_2_ (c).

### 3.8 Sorption Mechanism

pH effect, zeta potential measurement, and characterization studies provide important findings in explaining the possible interactions between MGEOP@MnO_2_ and cationic dye molecules. The high removal efficiencies observed over a wide pH range suggest that the process cannot be explained solely by electrostatic interaction between the cationic dye and the adsorbent surface, and that different interactions also play a synergistic role. At this point, it can be stated that –OH groups on the adsorbent surface and MnO_2_ nanoparticles participate in the process by forming hydrogen bonds with the amino groups in the dye structure.

## 4 Conclusion

This study proposes a straightforward approach for synthesizing geopolymer-based adsorbent and decorating it with metal oxides for effective decolorization. The results of this study demonstrate that MGEOP@MnO_2_ composite has an impressive sorption capability in both batch and dynamic flow treatment processes for removing BBY from an aqueous environment. The experimental data fit well with the pseudo-second-order and D-R models, according to the kinetic and isotherm modeling. The Langmuir model is also quite compatible with the decolorization process. The optimal batch adsorption variables for the BBY were identified using the Box-Behnken design tool to be pH 6.0, a dosage of 60 mg of MGEOP@MnO_2_ per 25 mL, and an adsorption capacity of 40.28 mg g^–1^. In the continuous system, 96.14% BBY was removed with 92.43 mg of adsorbent and a flow rate of 2.05 mL min^–1^. The outcomes proved that the MGEOP@MnO_2_’s predicted and experimental adsorption capacities are compatible. The adsorbent structure and possible interactions between BBY dye and MGEOP@MnO_2_ were clarified using BET, XRD, SEM-EDX, and FTIR analysis. It additionally emerged that MGEOP@MnO_2_ may successfully adsorb BBY dye from real wastewater. The combined effect of MnO_2_-coated geopolymer’s structural and chemical characteristics explains its better color removal efficiency. The MnO_2_ coating promotes metal oxides on the surface, while the high porosity and broad surface area of the geopolymer offer many adsorption sites for dye molecules. This increases the adsorption capacity by fortifying the contacts between the dye molecules and the adsorbent. In summary, this affordable and eco-friendly material is competitive with other adsorbents due to its greater surface area and pores, more active sorbent surface, and synergistic impact of MnO_2_ and geopolymer. Overall, this innovative sorbent offers a solid dynamic basis for environmental cleanup and serves as an effective and environmentally friendly option. The possibility of employing treatment systems coupled with MGEOP@MnO_2_ composites as part of a circular economy model for wastewater management is a fascinating subject for upcoming studies.

## Supplementary Material



## Notes

Supplementary tables including ANOVA results for the adsorption yield of BBY onto MnO_2_-coated geopolymer in batch and column systems (PDF)
