# Supplementary material for: A New Surface Decorated Geopolymer Matrix: Insights and Modeling into Comprehensive Sorptive Decolorization Applications
Source: ACS Omega. 2026 May 22;11(22):32636–51. doi: 10.1021/acsomega.6c01379 (PMC13261597; doi:10.1021/acsomega.6c01379)
Supplement: Supplementary file 1 [file ao6c01379_si_001.pdf]

SUPPLEMENTARY

A new surface decorated geopolymer matrix: insights and modeling into comprehensive sorptive decolorization applications

Sibel Tunali Akar[a*], Ilknur Kara[b], Ayse Yilmaz[c], Tamer Akar[a]

[a]*Eskişehir Osmangazi University, Faculty of Science*, *Department of Chemistry, 26040, Eskişehir, Türkiye*

[b]*Anadolu University, Faculty of Education, Department of Elementary Education, 26470, Eskişehir, Türkiye.*

[c]*Eskişehir Osmangazi University, Graduate School of Natural and Applied Sciences, Department of Chemistry, 26040, Eskişehir, Türkiye.*

**Corresponding author:* Sibel Tunali Akar

E–mail address: stunali@ogu.edu.tr (Sibel Tunali Akar)

Table S1 ANOVA results for the adsorption yield of BBY onto MnO_2-coated geopolymer in batch system

Variation source	Coefficients	Sum of squares	df	Mean square	F- Value	p-Value Prob>F
Intercept	97.37					
A	0.08	0.05	1	0.05	0.0036	0.9536
B	16.70	2231.94	1	2231.94	175.31	< 0.0001
C	7.01	393.02	1	393.02	30.87	0.0009
AB	1.33	7.11	1	7.11	0.56	0.4793
AC	0.89	3.17	1	3.17	0.25	0.6333
BC	−7.39	218.55	1	218.55	17.17	0.0043
A^2	−0.72	2.18	1	2.18	0.17	0.6912
B^2	−12.63	671.71	1	671.71	52.76	0.0002
C^2	−6.74	191.01	1	191.01	15.00	0.0061
Model		3770.41	9	418.93	32.91	< 0.0001
Residual		1.14	4	0.2839		
R^2 = 0.9769						
Adj. R^2 = 0.9472						

Table S2 ANOVA results for the adsorption yield of BBY onto MnO_2-coated geopolymer in continuous system

Variation source	Coefficients	Sum of squares	df	Mean square	F- Value	p-Value Prob>F
Intercept	57.50		1			
A	−8.93	637.80	1	637.80	65.41	< 0.0001
B	16.99	2310.24	1	2310.24	236.93	< 0.0001
C	−16.24	2109.02	1	2109.02	216.29	< 0.0001
AB	−2.68	28.66	1	28.66	2.94	0.1302
AC	−3.10	38.46	1	38.46	3.94	0.0874
BC	−0.53	1.11	1	1.11	0.1143	0.7452
A^2	3.34	46.89	1	46.89	4.81	0.0644
B^2	−8.12	277.55	1	277.55	28.46	0.0011
C^2	6.91	200.76	1	200.76	20.59	0.0027
Model		5625.29	9	625.03	64.10	< 0.0001
Residual		36.52	4	9.13		
R^2=0.9880						
Adj. R^2=0.9726						
